# Agitation and Dementia: Prevention and Treatment Strategies in Acute and Chronic Conditions

**DOI:** 10.3389/fneur.2021.644317

**Published:** 2021-04-16

**Authors:** Claudia Carrarini, Mirella Russo, Fedele Dono, Filomena Barbone, Marianna G. Rispoli, Laura Ferri, Martina Di Pietro, Anna Digiovanni, Paola Ajdinaj, Rino Speranza, Alberto Granzotto, Valerio Frazzini, Astrid Thomas, Andrea Pilotto, Alessandro Padovani, Marco Onofrj, Stefano L. Sensi, Laura Bonanni

**Affiliations:** ^1^Department of Neuroscience, Imaging and Clinical Sciences, University G. d'Annunzio of Chieti-Pescara, Chieti, Italy; ^2^Behavioral Neurology and Molecular Neurology Units, Center for Advanced Studies and Technology–CAST, University G. d'Annunzio of Chieti-Pescara, Chieti, Italy; ^3^Institute for Mind Impairments and Neurological Disorders–iMIND, University of California, Irvine, Irvine, CA, United States; ^4^Institut du Cerveau et de la Moelle épinière, ICM, INSERM UMRS 1127, CNRS UMR 7225, Pitié Salpêtrière Hospital, Paris, France; ^5^AP-HP, GH Pitie-Salpêtrière-Charles Foix, Epilepsy Unit and Neurophysiology Department, Paris, France; ^6^Neurology Unit, Department of Clinical and Experimental Sciences, University of Brescia, Brescia, Italy; ^7^Parkinson's Disease Rehabilitation Centre, FERB ONLUS–S. Isidoro Hospital, Trescore Balneario, Italy

**Keywords:** agitation, dementia, hyperkinetic delirium, Alzheimer's Disease, Dementia with Lewy Bodies, Frontotemporal Dementia, Vascular Dementia, COVID-19

## Abstract

Agitation is a behavioral syndrome characterized by increased, often undirected, motor activity, restlessness, aggressiveness, and emotional distress. According to several observations, agitation prevalence ranges from 30 to 50% in Alzheimer's disease, 30% in dementia with Lewy bodies, 40% in frontotemporal dementia, and 40% in vascular dementia (VaD). With an overall prevalence of about 30%, agitation is the third most common neuropsychiatric symptoms (NPS) in dementia, after apathy and depression, and it is even more frequent (80%) in residents of nursing homes. The pathophysiological mechanism underlying agitation is represented by a frontal lobe dysfunction, mostly involving the anterior cingulate cortex (ACC) and the orbitofrontal cortex (OFC), respectively, meaningful in selecting the salient stimuli and subsequent decision-making and behavioral reactions. Furthermore, increased sensitivity to noradrenergic signaling has been observed, possibly due to a frontal lobe up-regulation of adrenergic receptors, as a reaction to the depletion of noradrenergic neurons within the locus coeruleus (LC). Indeed, LC neurons mainly project toward the OFC and ACC. These observations may explain the abnormal reactivity to weak stimuli and the global arousal found in many patients who have dementia. Furthermore, agitation can be precipitated by several factors, e.g., the sunset or low lighted environments as in the sundown syndrome, hospitalization, the admission to nursing residencies, or changes in pharmacological regimens. In recent days, the global pandemic has increased agitation incidence among dementia patients and generated higher distress levels in patients and caregivers. Hence, given the increasing presence of this condition and its related burden on society and the health system, the present point of view aims at providing an extensive guide to facilitate the identification, prevention, and management of acute and chronic agitation in dementia patients.

## Introduction

Agitation is a common behavioral disturbance featuring exaggerated motor activity and verbal and/or physical aggressiveness, severe enough to impair social relations and daily living activities (1).

Agitation is observed in up to 70% of patients with cognitive decline, and its incidence is higher in moderate to severe stages of the disease (2). The prevalence ranges from 30 to 50% in Alzheimer's disease (AD), 30% in dementia with Lewy bodies (DLB), 40% in frontotemporal dementia (FTD), and 40% in vascular dementia (VaD) (3, 4).

Agitation adversely impacts cognitive performance, functional status, and patients' quality of life and enhances caregiver's distress. Moreover, agitation is associated with a higher admission rate to assisted living facilities, higher use of medications, long-term hospitalization, and higher mortality (5).

Several studies have considered agitation as indicative of the external expression of anxiety (6–8). Thus, some authors have suggested that the presence of anxiety in AD, a typical sign of the disease's preclinical stage, could also be seen as a risk factor for the future ensuing of agitation (9).

The occurrence of agitation is related to frontal lobe dysfunctions (10, 11) and, in particular, to an abnormal activation of the orbitofrontal cortex (OFC) and anterior cingulate cortex (ACC) (12). This notion has been supported by postmortem studies (12, 13) showing the large presence of neurofibrillary tangles in the OFC and ACC of patients with the frontal variant of AD. Indeed, a strict correlation among neuropsychiatric symptoms (NPS), especially aggressiveness, and tau pathology, rather than amyloid, is commonly reported in AD patients (12, 14, 15). A significant tau pathology in the frontal lobes is also a major postmortem finding in FTD, which is notably characterized by disabling behavioral changes (16, 17). As regards DLB and VaD, both characterized by NPS including agitation (18, 19), a clear correlation with frontal lobe dysfunction is lacking.

**Figure 1:**
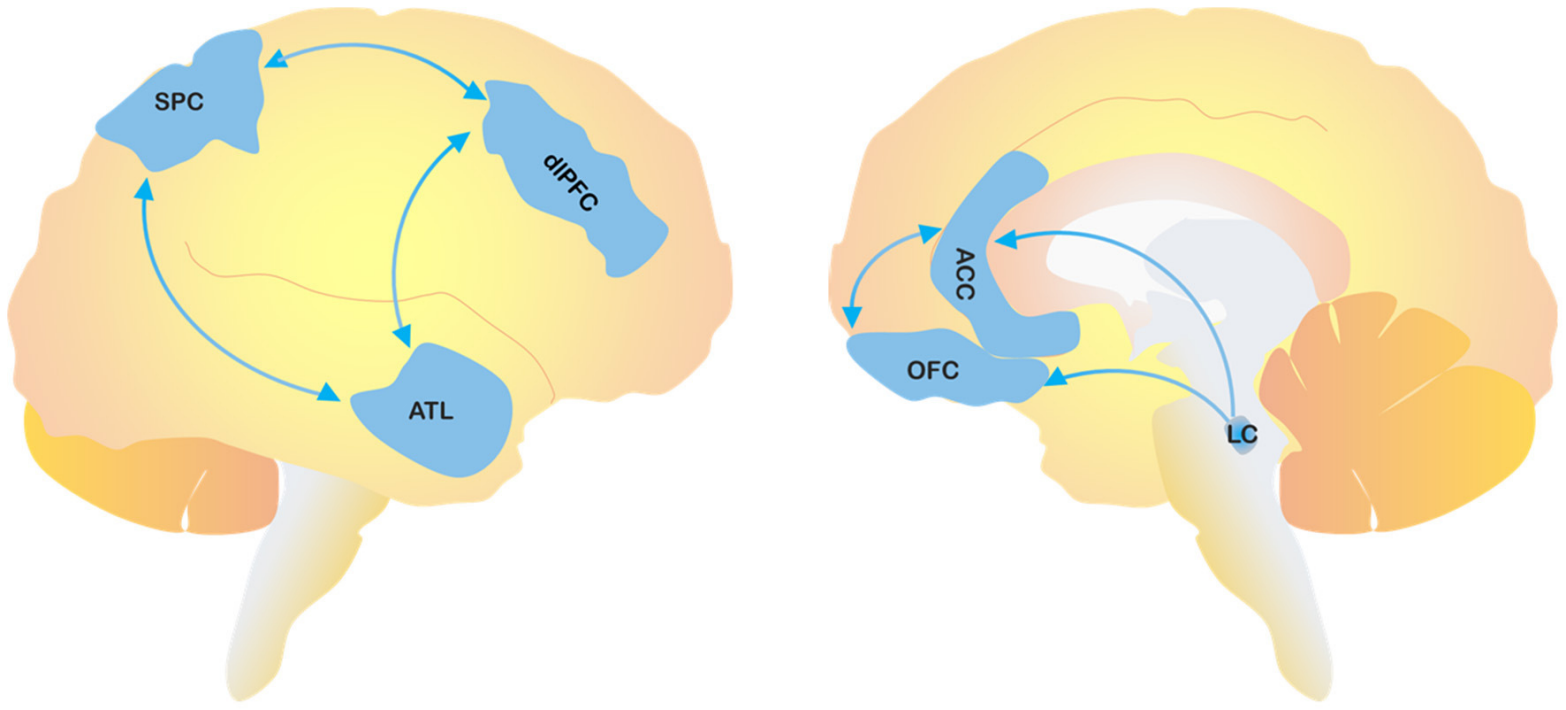
The figure depicts the cerebral areas involved in the pathological process of agitation. SPC, superior parietal cortex; dlPFC, dorsolateral pre-frontal cortex; ATL, anterior temporal lobe; OFC, orbitofrontal cortex; ACC, anterior cingulate cortex; LC, locus coeruleus.

The involvement of frontal lobe dysfunction in the occurrence of agitation has been also confirmed by functional imaging studies in both cognitively unimpaired subjects (20, 21) and AD patients (22). Furthermore, blood hypoperfusion in the anterior temporal lobe (ATL), dorsolateral pre-frontal cortex (dlPFC), and superior parietal cortex (SPC) is thought to contribute to the production of abnormal emotional responses to external stimuli, thereby causing aggressive or agitated states. The dlPFC is a critical area for thinking and planning, whereas the SPC is involved in sensorimotor integration from external stimuli. A dysfunctional interplay among these cerebral regions may explain abnormal behavioral responses driven by misinterpretation of environmental and social stimuli (23–26). Several studies have also supported the notion of imbalanced neurotransmitter systems in the onset of behavioral impairments (27). Besides the role of cholinergic depletion in AD and DLB, which may also be a mechanism underlying dysregulation of emotions and attention, as suggested by the efficacy of acetylcholinesterase inhibitors (AChEIs) in the control of psychiatric symptoms (28), the noradrenergic system also seems to be directly involved. A compensatory dysfunctional overactivity of noradrenaline, driven by the progressive loss of noradrenergic neurons in the locus coeruleus (LC), may affect the frontal cortex activity, thereby causing a regional up-regulation of adrenergic receptors (29, 30) (Figure 1). In FTD, even though the data are still controversial, agitation appears to be related to increased dopaminergic transmission (31).

Current managing guidelines for dementia-related agitation recommend implementing non-pharmacological approaches as the first line of intervention (32), whereas considerable research now focuses on prevention strategies. Pharmacological treatments should be initiated whenever behavioral changes may compromise patient safety and produce severe distress to caregivers (33).

The present review provides a toolbox for the detection, prevention, and therapeutical management of acute and chronic agitation in patients suffering from neurodegenerative conditions.

## Mild Behavioral Impairment

NPS are common in the prodromal stage of dementia and can precede the onset of cognitive impairment. The presence of NPS in cognitively normal patients or in patients with mild cognitive impairment (MCI) is associated with an increased risk of progression to overt dementia. The need to identify, in the early stages of the disease, the population at risk of cognitive decline has led to the formulation of the concept of mild behavioral impairment (MBI) (34). Building on the prior definitions of a pre-dementia risk state (35, 36) and frontotemporal-MCI (37), the ISTAART NPS-PIA formally described MBI as the emergence of sustained and impactful NPS occurring after the age of 50, which are not encompassed in the psychiatric nosology, persist for at least 6 months, and manifest before or at the onset of MCI (34). Among the NPS associated with MBI, agitation is as frequent as 30%. It is important to understand the prevalence of agitation and impulsivity in pre-dementia syndromes as there is a potential opportunity for early intervention and higher impact in this early stage of disease, even though clinical trials need to be conducted to test and prove that behavioral and pharmacologic treatments in the pre-dementia stage can effectively improve agitation.

## Pre-disposing Factors and Conditions to Agitation

Agitation occurs as a result of the interplay between neurobiological and environmental determinants. In addition to neurodegenerative processes, several factors, such as chronic or acute pain (e.g., arthritis), sleep disturbances, sensory impairment, acute medical illness (e.g., infections, respiratory diseases, urinary retention, renal failure), or metabolic changes (38), generate agitated behavior. Psychological factors, depression, or psychological distress may also be involved (39). Sundowning, the ensuing agitation upon the dimming of natural light as happens in the evening, is another predisposing condition for agitation (40). Moreover, acute onset of agitation can be triggered by changes in medication regimens or drug side effects (e.g., with antipsychotics or anticholinergic drugs) (40).

Hospitalization is another well-known trigger of agitation either alone or as a manifestation of hyperkinetic delirium in people with dementia (41).

Finally, severe acute respiratory syndrome coronavirus 2 (SARS-CoV-2) can be a precipitating factor for agitation because of the related long-term hospitalization and quarantine (42–44) or the ensuing viral encephalitis, critical illness encephalopathy, and systemic inflammation (45).

## Agitation in Acute Medical Conditions: Differential Diagnosis With Dementia-Related Agitation

In elderly patients, acute agitation can be the expression of a wide range of conditions, including medical, psychiatric, and substance-induced alterations (46) (Table 1).

**Table 1:** Differential diagnoses in case of dementia-related agitation and other conditions.

Neurological	• Stroke • CNS tumors • Intracranial hemorrhage • Meningitis • Encephalitis
Psychiatric	• Bipolar disorder • Schizophrenia • Delusions
Metabolic	• Electrolyte abnormalities • Hyperglycemia • Hypoglycemia
Toxicological	• Anticholinergic agents • Serotonergic agonists • Benzodiazepines • Steroids • Neuroleptics • Alcohol abuse • Alcohol withdrawal • Carbon monoxide toxicity
Infections	• Systemic infections • Fever-Related delirium • Sepsis

In addition to hyperkinetic delirium, which can also arise during the hospitalization of cognitively unimpaired older people (42), other acute neurological conditions, such as stroke (especially in the presence of aphasia), intracranial neoplastic masses or hemorrhages, meningitides, encephalitides, and head injuries, may generate agitation that can be the first or unique symptom of the underlying condition.

The ascertainment of a history of psychiatric illnesses (e.g., schizophrenia or other psychotic disorders, depression, bipolar disorders) is critical, as these conditions may often determine the onset of acute mental alterations, including agitated behavior (47).

Moreover, the presence of substance-induced agitation should always be considered. Older people are particularly susceptible to drug side effects, as co-existing metabolic disturbances can affect drug pharmacokinetics (40). Age-related metabolic alterations include hypoalbuminemia, hepatic or renal failure, and dehydration, conditions that should not be overlooked. Neurotransmitter alterations (e.g., a reduction in dopamine and acetylcholine transmission) can also promote acute agitation in elderly patients (48). Furthermore, the use of benzodiazepines (BZDs) may generate paradoxical response and behavioral disinhibition, as well as worsening of cognitive performances, excessive sedation, and increased risk of falls (49). Therefore, in older people, BZDs should be avoided, and their use or misuse should be taken into account in the event of an acute onset of agitated behavior. Anticholinergic and serotoninergic agonists, steroids, and neuroleptics (46, 50) can also trigger agitation in the elderly. Non-pharmacological toxic agents also need to be considered, especially in suspected carbon monoxide or alcohol intoxications. Substance withdrawal should also be considered as a possible trigger for agitation (48).

### Diagnostic Investigations

When dealing with a patient showing agitation signs, the primary goal is to identify cause and severity (51). A diagnostic workup is mandatory to achieve this goal.

The first step is to collect the patient medical history, ideally with caregivers' and relatives' critical help. To that aim, it is critical to identify the time of onset of agitation and the patient's pre-existing mental status. Recent hospitalizations, infections, traumas, or other predisposing factors, including medication history or substance abuse, should also be investigated (48, 51).

Accurate physical examination is key. Vital signs monitoring is mandatory to manage life-threatening conditions, such as cardiocirculatory or respiratory failure. The current mental status and level of consciousness of the patients should be assessed. Neck stiffness, fever, pain, or focal deficits should be evaluated to exclude infections (e.g., meningoencephalitis), stroke, hemorrhages, or central nervous system (CNS) tumors. Moreover, other abnormal findings, such as dehydration, asterixis, or vomiting, should prompt the investigation of underlying metabolic disorders or impairment (e.g., diabetes, renal, or liver failure) (46, 48).

All the agitated patients admitted to the hospital should be assessed with the help of laboratory testing (including the assessment of blood cell counts, glucose levels, electrolytes, creatinine, blood urea nitrogen, transaminases, C-reactive protein, procalcitonin, and urine tests) to rule out certain conditions, such as anemia, intercurrent infections, and liver or kidney failure. Toxicologic examinations should be considered to exclude alcohol or substance abuse. In acute agitation, a non-contrast head computed tomography (CT) should be obtained when an intracranial origin is suspected (48), especially in the presence of focal neurological signs. Lumbar puncture, as well as brain magnetic resonance imaging (MRI) and electroencephalogram (EEG), should be reserved for selected cases (e.g., when encephalitis, or a non-convulsive status epilepticus, is suspected) (48, 52).

### Assessment Scales for Agitation

Several assessment scales are currently available to investigate the presence and severity of agitation (53). The Agitated Behavior Scale (ABS) assesses an agitated state's occurrence and duration after brain injury. The scale's primary purpose is to monitor behavioral changes after admission to a hospital ward (54). The Behavioral Activity Rating Scale (BARS) is often used in clinical trials. According to this scale, patients are classified into seven different levels of agitation (55). The Overt Agitation Severity Scale (OASS) offers a helpful approach to detect and rate agitation when spanning from anxiety to aggression (56). The Modified Overt Aggression Scale (MOAS), a scale divided into four sections, examines the frequency and severity of aggressive episodes (57). Another easy-to-use tool is the Pittsburgh Agitation Scale (PAS), which measures the dementia-related agitation severity (58). The Agitated Behavior Mapping Instrument (ABMI) (59) is a diagnostic scale that evaluates 14 different physical and verbal agitated behavior features. Finally, the Cohen-Mansfield Agitation Inventory (CMAI) is a frequency rating scale completed by caregivers (60).

Some assessment scales are primarily administered in high-intensity care wards. The Riker Sedation–Agitation Scale (SAS) (61), the first specific agitation scale developed for the intensive care unit (ICU), identifies seven levels of consciousness ranging from severe and life-threatening agitation to deep sedation. A similar tool is the Motor Activity Assessment Scale (MAAS) (62), which describes, according to the patient's motor behavior, seven different levels of agitation (from unresponsive to dangerously agitated). The Richmond Agitation Sedation Scale (RASS) assesses the level of alertness and agitated behavior in critically ill patients (63).

A screening tool for delirium is the 4AT, in which a score higher than four indicates a high risk for delirium (64). However, the Confusion Assessment Method for the ICU (CAM-ICU) is a more sensitive and specific assessment to diagnose a delirium state (65).

## Managing Agitation With Non-pharmacological Strategies

Over the past years, several studies have investigated a wide range of non-pharmacological approaches to treat and prevent dementia-related agitation. Hereby, we discuss the most validated first-line non-pharmacological approaches to chronic and acute agitation in cognitively impaired subjects (32, 66).

### Prevention Strategies for Home-Living and Care Home Residents

Person-centered care (PCC) is an operating system in healthcare, which considers the health practitioner and the patients as partners in achieving tailored care that meets patients' needs in a unique way (67, 68). In the PCC framework, the social and historical background, the personality, and the lifestyle of the patients are considered to promote a positive social environment, good compliance, and best outcomes for patients with dementia (67, 68). The PCC approach is considered a successful option to prevent agitation in home-living and care home patients with dementia (69, 70) and reduce antipsychotic use (71). Nonetheless, not every PCC-based strategy is effective. For instance, the Dementia Care Mapping (DCM) (69, 72, 73), which is based on patients' systematic observations, has generated conflicting results. In contrast, the Managing Agitation and Raising Quality of Life (MARQUE) intervention (74) has failed to improve agitation prevention in care home settings.

As indicated by a recent meta-analysis (75), music intervention, especially when employed in groups, can significantly reduce agitation in cognitively impaired patients. Furthermore, passive listening to music has been associated with behavioral improvement and reduction of aggressiveness and agitation (76). This effect is considered to be driven by a positive exploration of repressed feelings (76). Regarding music therapy trials, some studies have explored the effectiveness of white noise to prevent the sundowning syndrome, or the use of personalized music, *via* headphones, upon daily hygiene care or walking, reporting that these approaches can improve agitated behavior (77). Doll therapy has been shown to improve happiness and engagement but did not significantly reduce agitation (78, 79). Some concerns have been raised on this approach's feasibility in patients who do not have offspring (79). Thus, further investigation and research are required.

Previous studies have indicated a role for boredom in the onset of physical agitation (80). Thus, activity-based strategies have been developed to prevent this condition. The application of combined stimuli (tasks, reading, work-related, manipulation) has shown to be more effective than a single activity (81). However, the most effective activity was live socialization (82).

New technologies, which still need full validation, are being developed to monitor and prevent agitation. Behavioral & Environmental Sensing and Intervention (BESI) collects environmental data, caregivers' information, and patients' behavioral and motion states through wearable tools and then detects early signals of incoming agitation delivering an alert to the caregiver, who can timely prevent the complete escalation of the symptom (83–85). A teleconsultation system based on expert's recommendations, namely, ECHO-AGE, has also provided clinical improvement in nursing home settings when dealing with agitated patients (86).

Several other non-pharmacological interventions are being evaluated for agitation prevention, as virtual and sensory therapy (e.g., aromatherapy, bright light therapy, taking a breath of fresh air, the use of rocking chairs) (77, 79). Lastly, ongoing studies are proposing virtual reality-related approaches that include music, sensory, and mental stimulations to improve cognitive functions (attention, executive function, visual and verbal memory) and psychological domains (agitation, depression, anxiety, and apathy) (87–89).

Data that emerged from other non-pharmacological approaches, such as acupuncture and electroconvulsive therapy, are mostly conflicting. Some ongoing trials could possibly clarify a potential role for these therapies (90, 91).

### Therapeutic Strategies for Hospitalized Patients

Unfortunately, the PCC approach is challenging in hospital settings, given the short length of the patient stay and the necessity of rapid interventions (92). However, the presence of pain (93) and intercurrent pathologic conditions (94) (e.g., infections, dehydration, metabolic imbalance) has been associated with the onset of aggressiveness, anxiety, and agitation and should be addressed. Furthermore, the direct assessment of unmet needs in hospitalized patients, which is often less recognized and identified than expected, can provide a more time-effective approach in a clinical environment, according to the standards of “Needs-driven care” (68) based on the Unmet Needs Model (80). Nonetheless, cognitively impaired patients may experience difficulties communicating feelings of discomfort, pain, and loneliness, thereby making the assessment difficult (80). In nursing homes or at the patient home, the Needs-driven care is ancillary to the PCC and the most successful given the presence of familiarity and closed relationships with the patients.

Moreover, as previously reported, patients with dementia, when hospitalized, often present with a more complex clinical picture of hyperkinetic delirium (42). Combined strategies are far more adequate to prevent delirium onset, yet not always effective (95). Careful management of pharmacological therapy, space–time reorientation, early mobilization, minimization of restraint use, and adequate sleep hygiene is the most recommended option for preventing delirium in hospitalized patients (42, 96). Other options include access to a living room with other patients and the caregiver's presence (97). Multiple changes of rooms should be avoided (98).

All the strategies developed in the last 20 years have led to a sophisticated solution, the “Delirium Room” (DR) (99, 100). Elderly patients with cognitive decline and the need for intensive/continuous observation can be monitored, in this dedicated four-bed environment, by a trained nurse. Restraint use is strongly discouraged, whereas non-pharmacological approaches to prevent agitation are indicated (99). A valid option is offered by the Geriatric Monitoring Unit (GMU) (101, 102). The GMU combines, in a five-bed room, typical DR management with a PCC approach and bright therapy and has been shown to achieve significantly shorter lengths of delirium symptoms (102).

## Pharmacological Treatments in Acute and Chronic Conditions

The Food and Drug Administration (FDA) warning on the use of antipsychotic drugs (both typical and atypical) has led to better awareness about the risk associated with the use of these classes of drugs in cognitively impaired and older people, including increased mortality (103–105).

The recently published European Academy of Neurology (EAN) recommendation on the management of issues in dementia, including agitation, reports that individuals with dementia and agitation and/or aggression should be treated with atypical antipsychotics only after all non-pharmacological measures have been proven to be without benefit or in the case of severe self-harm or harm to others (weak recommendation). Antipsychotics should be discontinued after cessation of behavioral disturbances and in patients in whom there are side effects (Good Practice statement) (66).

Further suggestions for pharmacological management of acute agitation, such as agitation in delirium and chronic agitation in dementia, are depicted in the following sections. Randomized controlled trials (RCTs) are summarized in Table 2.

**Table 2:** Summary of randomized, controlled trials (RCTs) for agitation in dementia.

**Drug**	**RCT**	**Endpoints**	**Disease**	**Clinical findings**
Citalopram and perphenazine (106)	Placebo-controlled trial	Efficacy for the treatment of psychosis and behavioral disturbances	Non-depressed hospitalized patients with dementia	Significant improvement on agitation. Compared with placebo, only citalopram was found to be more efficacious in the short-term hospital treatment of psychotic symptoms
Citalopram and risperidone (107)	Not vs. placebo	Efficacy of risperidone and citalopram for psychotic symptoms and agitation, respectively Second endpoint: citalopram would be associated with fewer side effects	Non-depressed hospitalized patients with dementia	Agitation and psychotic symptoms decreased in both treatment groups without significant differences. Citalopram was associated with fewer side effects
Citalopram (108)	Placebo-controlled trial	Efficacy of citalopram for agitation Second endpoints: citalopram effects on function, caregiver distress, safety, cognitive safety, and tolerability	AD	Citalopram reduced agitation and caregiver distress. However, cognitive and cardiac adverse effects of citalopram may limit its practical application at the dosage of 30 mg/day
Citalopram (109)	Comparison with quetiapine and olanzapine	Efficacy and safety of citalopram	Nursing home residents with AD	Citalopram resulted in similar efficacy and less adverse effects when compared with the two atypical antipsychotics
Escitalopram (110)	Comparison with risperidone	Efficacy and tolerability of escitalopram	Hospitalized patients with AD	Escitalopram and risperidone did not differ in efficacy
Sertraline (111)	Placebo-controlled trial	Efficacy of sertraline	Non-depressed patients with severe probable AD	A decreased aggression trend with sertraline
Mirtazapine (112)	Not vs. placebo	Effectiveness and safety of mirtazapine	AD	Mirtazapine showed its efficacy for treatment of agitated patients with AD. There were not significant side effects and cognitive deterioration
Memantine (113)	Placebo-controlled trial	Efficacy of memantine	Patients with moderate to severe AD	Memantine did not show its effectiveness compared with placebo
Memantine (114)	Placebo-controlled trial Comparison with antipsychotics	Efficacy of memantine compared with antipsychotics Second endpoints: improvement in NPI, MMSE, and mortality	AD	No benefits for memantine in the long-term treatment and prophylaxis
Carbamazepine (115)	Placebo-controlled trial	Efficacy of carbamazepine	Nursing home residents with dementia	Clinical global improvement
Oxcarbazepine (116)	Placebo-controlled trial	Efficacy of oxcarbazepine	Institutionalized patients with AD or VaD	No significant effects of oxcarbazepine
Quetiapine (117)	Placebo-controlled trial	Efficacy and tolerability of quetiapine	DLB and PDD	Quetiapine was well-tolerated and did not worsen parkinsonism
Rivastigmine (118)	Placebo-controlled trial	Efficacy of rivastigmine	DLB	Rivastigmine 6–12 mg daily produced statistically and clinically significant behavioral effects
Rivastigmine (119)	Placebo-controlled trial	Efficacy of rivastigmine	PDD	Rivastigmine was associated with moderate improvements in agitation but also with higher rates of nausea, vomiting, and tremor
Donepezil (120)	Placebo-controlled trial	Safety and efficacy of donepezil	PDD	Donepezil improved cognition and seemed to be well-tolerated and not to worsen parkinsonism
Donepezil (121)	Placebo-controlled trial	Safety and efficacy of donepezil	PDD	Beneficial effects on memory and improvement of other cognitive deficits
Donepezil (122)	Placebo-controlled trial	Safety and efficacy of donepezil	PDD	Donepezil was well-tolerated and did not worsen PD. A modest benefit on cognitive function was observed
Trazodone (123)	Placebo-controlled trial	Efficacy of trazodone	FTD	Trazodone is an effective treatment for the behavioral symptoms of FTD
Rivastigmine (124)	Placebo-controlled trial	Efficacy, safety, and tolerability of rivastigmine capsules	VaD	Rivastigmine did not provide consistent efficacy. The efficacy on cognitive outcomes was derived from effects in older patients likely to have concomitant Alzheimer pathology
Pimavanserin (NCT03325556) (125)	Placebo-controlled trial Comparison with 20 or 34 mg of pimavanserin to placebo	Efficacy of pimavanserin	Dementia-related psychosis	Ongoing (phase 3)
Lumateperone (NCT02817906) (126)	Placebo-controlled trial	Efficacy and safety of lumateperone administered orally once daily in the treatment	Demented patients, including AD	Failed
Brexpiprazole (NCT03548584) (127)	Placebo-controlled trial	Efficacy, safety, and tolerability of two doses of brexpiprazole	AD	Ongoing (phase 3)
Brexpiprazole (NCT03724942) (128)	Placebo-controlled trial	Safety of brexpiprazole 1 or 2 mg after a 14-week treatment regimen for agitation associated with AD patients who completed a double-blind trial, and to investigate the efficacy of brexpiprazole	AD	Ongoing (phase 3)
Brexpiprazole (NCT03620981) (129)	Placebo-controlled trial	To evaluate the superiority of brexpiprazole 1 or 2 mg over placebo after a 10-week treatment regimen, to investigate its safety and to identify the optimum dose	AD	Ongoing (phase 3)
Dextromethorphan/quinidine (AVP-923) (130)	Placebo-controlled trial	Safety, tolerability, and efficacy of AVP-923	AD	Significant effects in reducing agitation
Dextromethorphan (AVP-786) (131)	Placebo-controlled trial	Safety, tolerability, and efficacy of AVP-786	AD	Ongoing (phase 3)
Cannabis (132)	Placebo-controlled trial	Efficacy of cannabis for agitation Second endpoints: reduction in medication consumption, weight gain, and improvement of sleep	Demented patients	Ongoing (phase 2)
Nabilone (NCT02351882) (133)	Placebo-controlled trial	Safety and efficacy of Nabilone	AD	Beneficial effects, although patients reported significant sedation

*AD, Alzheimer's disease; DLB, dementia with Lewy bodies; PDD, Parkinson's disease with dementia; VaD, vascular dementia; FTD, frontotemporal dementia; MMSE, Mini-Mental State Examination; NPI, Neuropsychiatric Inventory*.

**Figure 2:**
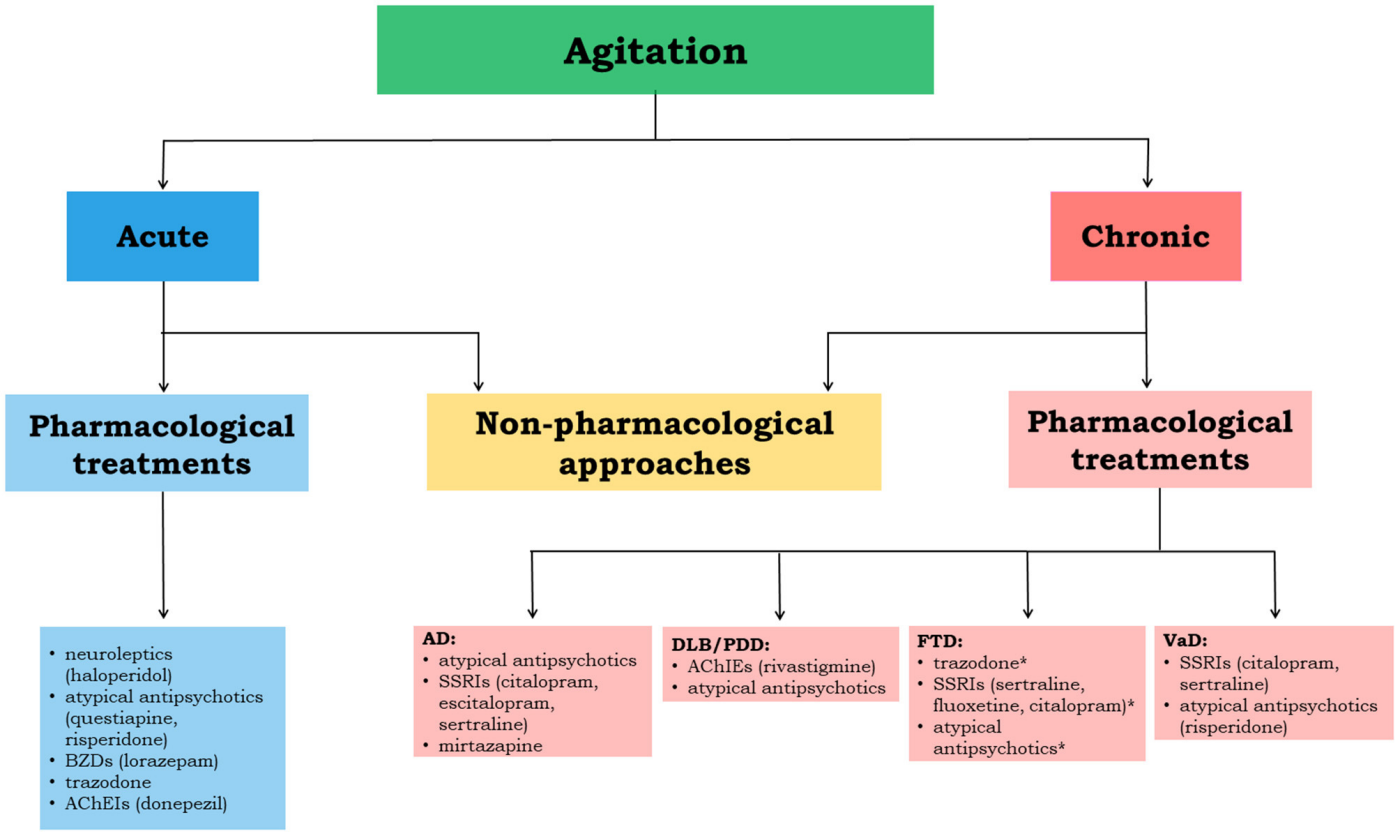
The flowchart depicts the therapeutical management flow to be implemented in acute and chronic agitation. The most relevant evidence-based drug options are reported for each neurodegenerative disease. *indicates not sufficiently supported by clinical evidence; AD, Alzheimer's disease; DLB, dementia with Lewy bodies; PDD, Parkinson's disease with dementia; VaD, vascular dementia; FTD, frontotemporal dementia; AChEIs, acetylcholinesterase inhibitors; BZDs, benzodiazepines; SSRIs, selective serotonin reuptake inhibitors.

In Figure 2, we propose an approach to treat patients with agitation properly.

### Agitation in Delirium

Any pharmacological treatment for agitation in hyperkinetic delirium should be started at the lowest dose and continued for the minimum amount of time (134).

The first step is to consider the patient medical history, including concomitant therapies and comorbidity (135), in order to reduce/minimize the underlying causative factors. In case of an agitated state, neuroleptics represent the first choice, especially haloperidol (starting dose ranging from 0.5 to 1 mg, not to exceed from 3 to 5 mg in 24 h), which also presents the advantage of parenteral formulation (136, 137). Given the drug's well-known parkinsonian side effects, it should be avoided in patients with dementia associated with parkinsonism (138). Indeed, in patients with extrapyramidal signs, pharmacological recommendations suggest using atypical antipsychotics, such as quetiapine (25–50 mg/day) (135). Three previous RCTs showed comparable clinical efficacy of atypical antipsychotics and haloperidol (137, 139, 140). Moreover, except for chlorpromazine, first- and second-generation antipsychotics can be safely administered in case of liver or kidney dysfunction (141).

In general, both typical and atypical antipsychotics should be avoided in patients with known cardiac disease due to pro-arrhythmogenic effects (e.g., QT interval prolongation) (142). In this case, BDZs, *per os* or *via* intramuscular administration according to delirium severity, should be preferred in patients without respiratory deficits or in the delirium/agitation associated with alcohol withdrawal (143, 144). Among BDZs, lorazepam (0.5–1 mg *per os*) seems to be the most suitable drug due to its pharmacokinetic properties (it is directly metabolized through glucuronidation, which potentially reduces drug interactions) (145). When treating patients with dementia and hyperkinetic delirium, it is important to consider the known possible paradoxical effects of BDZs with increased agitation in these patients (49).

Cognitively impaired patients with history of seizures or chronic pain can also be treated with antiepileptic drugs in case of mild delirium (135). Among anticonvulsant drugs, gabapentin and pregabalin present a good safety profile, even if they should be avoided in the presence of severe renal failure (146).

Some findings also supported the use of low doses of trazodone (50–300 mg/day) in treating hyperkinetic delirium (147–149). Indeed, this drug mainly acts through direct serotonin receptor blockade and may produce sedation due to its strong histaminergic effects (149).

Finally, some case reports and one open-label study (150–153) suggested a promising use of AChEIs, such as donepezil (5 mg daily), in delirium treatment, but further evidence and RCTs are needed.

### Alzheimer's Disease

Among typical antipsychotics, haloperidol in a dose of 1.2–3.5 mg/day suppresses aggressiveness effectively but shows lower efficacy on agitation. Because of its remarkable side effects (e.g., extrapyramidal signs, prolongation of the QTc interval, arrhythmias, and increased mortality), haloperidol is not recommended (142). Atypical antipsychotics show comparable effectiveness and higher patient tolerance. For instance, in a multicenter, double-blind, placebo-controlled trial, 421 outpatients suffering from AD with psychosis, aggressiveness, or agitation were randomly assigned to receive olanzapine (mean dose 5.5 mg/day), quetiapine (mean dose 56.5 mg/day), risperidone (mean dose 1.0 mg/day), or placebo. Clinical benefits were observed in 32% of patients assigned to olanzapine, 26% of patients assigned to quetiapine, 29% of patients assigned to risperidone, and 21% of patients assigned to placebo, without significant differences (154). The CATIE-AD study evaluated the effects on NPS of olanzapine, risperidone, and quetiapine, compared with placebo. Among NPS, antipsychotic resulted to be more effective for specific behavioral symptoms, such as agitation (155). Regarding second-generation antipsychotics, high doses are not recommended as the risk of mortality is dose-dependent. The most common causes of death are cardiovascular, cerebrovascular, respiratory, and infectious (especially respiratory) complications (142).

Moreover, selective serotonin reuptake inhibitors (SSRIs), such as citalopram, escitalopram, and sertraline, have demonstrated efficacy in AD patients (111, 156). The use of citalopram is supported by the most compelling evidence for treating AD dementia-related agitation (156). Despite earlier studies not focusing on agitation effects, in the first RCT, citalopram and perphenazine were more effective than placebo in short-term hospital treatments of psychotic disturbances, including agitation (106). Another RCT also demonstrated that citalopram decreases agitation scores compared with risperidone, without relevant side effects (107). Additionally, the Citalopram for Agitation in Alzheimer Disease Study (CitAD), a placebo-controlled double-blind RCT, showed a high acceptability rate and efficacy for citalopram over placebo in the treatment of agitation (108). In a nursing home setting, a longitudinal 6-month study showed similar efficacy against agitation of citalopram, olanzapine, or quetiapine, even though citalopram was associated with a lower occurrence of falls, orthostatic hypotension, and fewer hospitalizations (109). Citalopram should be considered in AD patients below 85 and used at low doses as cardiac conduction disturbances are dose-dependent and more frequent in elderly patients. The FDA recommends a maximum daily dose of citalopram of 20 mg/day in patients above the age of 60 (157). Since the effects of citalopram take 2 weeks to ensue, citalopram should not be considered for the acute treatment of agitation (156).

Escitalopram also showed clinical benefits in agitation treatment (158). For instance, a 6-week RCT compared escitalopram to risperidone and showed that both drugs reduced agitation. Although risperidone revealed efficacy earlier, the drug produced a higher burden of side effects (110). Since escitalopram may also prolong the QT interval in a dose-dependent manner, a low dose should be considered in the elderly (156). In that respect, the maximum daily dose recommended is 10 mg for patients over 60 (159).

Furthermore, sertraline was demonstrated to decrease agitation or aggressiveness and other psychiatric symptoms, in a double-blind, randomized, placebo-controlled study (111). In particular, AD patients with moderate to severe behavioral symptoms seem to respond to sertraline (156).

Mirtazapine, an antidepressant with alpha-2 adrenergic, 5-HT2, and 3-HT2 antagonist properties, showed promising results in open-label studies. For instance, in a 12-week prospective study, AD patients with agitated behavior receiving 15–30 mg/day of mirtazapine showed a significant reduction in CMAI-short form (CMAI-SF) after treatment (112).

Even if AChEIs seem to have positive effects on other behavioral symptoms (such as depression and anxiety) (142), they did not show good efficacy in the treatment of agitation in AD (160–162). Despite the initial positive results of memantine for the treatment of agitation and psychosis in AD (163), two subsequent RCTs failed to achieve statistically significant results in agitation (113, 114).

Small, placebo-controlled studies on carbamazepine found evidence of modest benefits for agitation and aggression in AD patients (115). Moreover, in 103 institutionalized cognitively impaired patients with agitation and aggressiveness, oxcarbazepine did not improve NPS vs. placebo, and side effects occurred more frequently in the treatment group (116). Regarding valproate (daily dose ranging from 480 to 1,500 mg/day), RCTs and subsequent meta-analysis failed to provide any support for its use in agitation (115). Few data, including mainly case reports and small case series, are available for other anticonvulsants (gabapentin, levetiracetam, topiramate, and lamotrigine) (115).

Prazosin, an alpha-adrenoreceptor blocking drug, demonstrated a significant impact on agitation and aggressiveness in a daily dose ranging from 1 to 6 mg (164), but it is not recommended in patients who have experienced orthostatic hypotension (165).

### Vascular Dementia

VaD is a neurologic condition due to the occurrence of single or multiple ischemic lesions of the brain (166). However, risk factors for cerebrovascular injuries also contribute to neurodegenerative disease onset. Rather than a “stand-alone” entity, VaD should be regarded as part of a “Mixed dementia” phenotype, particularly in elderly subjects (167). In this perspective, we can easily understand why RCTs have a hard time in disentangling VaD from AD (the most common cause of neurodegenerative dementia). Considering this limitation, as well as the lack of recent studies addressing the issue of agitation in VaD, we report the most relevant approaches identified.

A sequential drug treatment algorithm for agitation and aggressiveness associated with AD and mixed dementia, such as AD/VaD, has been lately proposed (168). The authors indicated that antipsychotic drugs have the highest efficacy for treating behavioral and psychotic symptoms. However, the risk of well-known side effects limits their use to only severe conditions (33).

Among antipsychotic drugs, risperidone seems to present the most substantial benefit for agitation, as well as other behavioral changes (169–171). In a clinical trial comparing dosage and time needed to reach a therapeutic response of three antipsychotics (risperidone, sulpiride, and quetiapine), the risperidone-treated group showed the shortest time to obtain a clinical response (172). The authors recommended a target dose ranging from 0.5 to 1.5 mg/day (173). Despite limited findings, also aripiprazole and quetiapine showed statistically significant effects on agitation (170, 174).

Some meta-analyses also revealed clinical benefits on agitation using SSRIs, especially citalopram and sertraline (175). AChEIs failed to improve the cognitive functions and behavioral symptoms of VaD patients (176–178). In a double-blind study, only rivastigmine showed its superior efficacy over placebo on cognition, but effects were missing when dealing with daily living activities or NPS (124).

Anticonvulsants, such as carbamazepine, gabapentin, and pregabalin, may be useful in patients who did not respond to antipsychotics, although clear evidence is lacking (118–120, 179).

### Dementia With Lewy Bodies and Parkinson's Disease With Dementia

The first step in treating agitation of parkinsonian patients should be an accurate revision of current treatment. Dopamine agonists (DAs), monoamine oxidase inhibitors (MAOIs), and catechol-O-methyltransferase (COMT) inhibitors, especially when associated with SSRIs or serotonin and norepinephrine reuptake inhibitors (SNRIs), might increase agitation (180). Thus, careful reduction or redistribution of dopaminergic drugs should represent the first option for these patients.

The management of agitation in DLB and Parkinson's disease with dementia (PDD) is particularly challenging in light of the well-documented side effects of typical and, to a lesser extent, atypical neuroleptics, compounds that worsen parkinsonism and even precipitate the ensuing of the neuroleptic malignant syndrome (181–184). Hence, the treatment with neuroleptics should be avoided in agitated patients with DLB/PDD. Nevertheless, some anecdotal evidence has indicated the efficacy and safety of atypical neuroleptics. In particular, clozapine (average dose 44.6 mg/day) (185) and quetiapine (50–75 mg/day) (117), atypical neuroleptics with weak affinity for dopamine D2 receptors, have achieved benefits in the management of agitation in patients with dementia and parkinsonism (117, 185). However, in line with the recent concerns about an increased risk of death in elderly patients treated with antipsychotic drugs (both atypical and conventional) (186), clozapine and quetiapine should be cautiously employed as a first-line treatment for agitation. AChEIs (118, 179) show modest efficacy in improving the cognitive and behavioral features of DLB/PDD patients and lack the risk of triggering extrapyramidal effects, although increased tremor is sometimes limiting. A sizeable placebo-controlled study on rivastigmine found that the use of rivastigmine in PDD patients significantly decreased the NPS, although an improvement of agitation was not clearly reported (119). Three placebo-controlled studies on donepezil in PDD patients (120–122) produced no effects vs. placebo for psychiatric disturbances.

### Frontotemporal Dementia

No FDA-approved therapies are available for FTD (174). Nevertheless, clinicians often liberally implement drugs used in other forms of dementia, even in the absence of evidence-based support. For instance, AChEIs and memantine failed to provide benefits in FTD patients and actually exacerbated behavioral symptoms (187–189).

Alterations of serotoninergic and dopaminergic rather than cholinergic neurotransmission are present in FTD (190), and therefore, SSRIs and antipsychotic drugs have been tested with different results. The clinical effects of sertraline, fluoxetine, and citalopram are controversial. These drugs reduced agitation, anxiety, and impulsiveness and worsened apathy and cognitive functioning (191–193). Some evidence suggested that SSRIs may help treat some behavioral features without affecting cognition, but further studies are needed (187). Trazodone has also been tested in clinical trials, and a study has supported its effectiveness against agitation and other NPS (188). In a randomized, placebo-controlled trial, trazodone (with daily doses ranging from 50 to 300 mg) dramatically reduced behavioral symptoms over 12 weeks of treatment. Common mild side effects included fatigue, dizziness, hypotension, and somnolence (123). Nevertheless, there is insufficient evidence to support the employment of trazodone for agitation in patients with FTD (148).

Among antipsychotic agents, risperidone, quetiapine, olanzapine, aripiprazole, and clozapine showed efficacy on the agitation of FTD patients, but, because of their low safety profile, the drugs are recommended only as a second-line treatment (194–196).

Recently, a low oral dose of lithium was proposed as an add-on therapy to antipsychotic treatment for aggressiveness and agitation in FTD (197). However, only three patients with FTD and three patients with AD were included in the study, and therefore, further RCTs are needed.

Regarding the use of antiepileptic drugs, two studies tested gabapentin for acute onset of agitation, but not a large and robust body of evidence is available to suggest its use in FTD patients (198).

## COVID-19 and Agitation in Neurodegenerative Diseases

After the first cases of the novel coronavirus disease 2019 (COVID-19) were reported in Wuhan, China, in December 2019, the spread of SARS-CoV-2 rapidly became a pandemic, involving millions of patients worldwide. Although SARS-CoV-2 poses a risk at all ages, adults aged 65 years and older are at greatest risk of severe disease, hospitalization, intensive care use, and death, accounting for more than 80% of deaths in Western countries. With the increasing number of confirmed cases and the accrued clinical data, it is now well-established that a significant proportion of these patients experience neurological symptoms and syndromes (in addition to the pre-dominant respiratory symptoms) (199, 200). Early COVID-19 studies have reported estimated rates of delirium at 20–50% and agitation at almost 10% at in hospitalized patients with dementia, even in the absence of typical signs and symptoms of SARS-CoV-2 infection, such as fever or cough (201). Different studies have clearly reported that the presence of delirium and/or agitation was associated with increased mortality, even taking into account age and multimorbidity (200, 202, 203).

Acute agitation in patients with delirium evoked by hypoxia and/or fever is the most commonly observed clinical feature, especially in elderly individuals with pre-existing dementia or psychiatric conditions (204). Given the elevated rates of clinical and adverse events associated with dementia and the various NPS potentially associated with COVID-19, physicians need to be mindful of these potential complications when evaluating these patients. A thorough examination of mental and neurological status helps the clinicians evaluate any acute behavioral disorder, which may indicate underlying encephalopathies or encephalitis triggered by the COVID-19 infection (199, 205).

Hospitalized patients with COVID-19, who are acutely agitated, deserve special attention given the risk of poor compliance to mechanical ventilation. Furthermore, the implementation of non-pharmacological and reorientation strategies to treat agitation is compromised by social distancing and isolation measures implemented to minimize the pandemic (206). The physical isolation of COVID-19 patients, either hospitalized or not, could trigger agitation and is still a critical issue in acute and chronic care. Patients with persistent and severe agitation despite attempts to treat the underlying causes require sedative medications. Low doses of first-generation antipsychotics, such as haloperidol, or second-generation antipsychotics, such as olanzapine and risperidone, are equally effective and are considered first-line management strategies in COVID-19 units (103, 207). However, no RCTs have addressed the treatment of COVID-19-related agitation (207). When there are no absolute contraindications to any particular medication class, possible management includes administering alpha-2 agonists and low-potency antipsychotics, whereas BDZs should be avoided given their depressive effects on ventilation.

Finally, the occurrence of hyperkinetic delirium and agitation has been reported also during the recovery phase from COVID 19, in the absence of direct brain infection (208).

## Clinical Pharmacological Trials and Future Prospects

Nowadays, dementia-related agitation treatments pose a significant challenge to clinicians as specific pharmacological therapies are lacking.

Several experts have agreed that, to optimize the clinical response, patients with dementia should be treated with specific and novel medications interacting with pharmacologically relevant targets (209–211). Therefore, preclinical and clinical studies focused on novel biological targets are needed.

Considering that the serotonin 5-HT2AR polymorphisms and altered functioning of these receptors are associated with psychosis and aggressiveness onset in AD patients, recent studies have been focused on the effects of pimavanserin, a selective inverse agonist of the 5-HT2AR (212) or lumateperone and brexpiprazole, two novel atypical antipsychotics acting as 5-HT2A antagonists. After showing promising efficacy in phase II RCTs, these drugs now face validation through phase III RCTs (213–215).

Regarding pimavanserin, recent phase II RCTs suggested efficacy in AD patients, although the safety profile needs further investigations. For instance, the drug may induce QT prolongation (216). A phase III, double-blind, placebo-controlled, relapse prevention study (NCT03325556) is evaluating the efficacy and safety profile of variable dosages of pimavanserin (125, 216).

Brexpiprazole is a second-generation antipsychotic, occasionally referred to as a third-generation antipsychotic with partial agonist properties at the D2 receptor. The compound is FDA-approved for treating schizophrenia and major depressive disorder as add-on therapy (217). Compared with aripiprazole and brexpiprazole, the molecule shows less activity at the D2 receptor, thereby generating a more tolerable side effects profile. Three phase III clinical trials are ongoing to evaluate the compound's long-term safety and clinical efficacy in AD patients with agitation (NCT03724942, NCT03620981, and NCT03548584) (127–129).

A recent phase III, randomized, double-blind, placebo-controlled, multicenter study (NCT02817906) investigating the efficacy of lumateperone in reducing dementia-related agitation failed (126).

Moreover, molecules acting *via* the inhibition of serotonin transporter (SERT) are another class of therapeutic options (218, 219). Dextromethorphan seems to be the most promising because of its high affinity to SERT. To improve its unfavorable pharmacokinetics, dextromethorphan has been combined with a cytochrome P450 2D6 inhibitor, quinidine (220). Quinidine reversibly inhibits cytochrome P450 2D6, thereby prolonging dextromethorphan plasma half-life. The first clinical study, a phase II, randomized, multicenter, double-blind, placebo-controlled trial (NCT01584440), evaluated the efficacy of AVP-923 (dextromethorphan/quinidine) at doses ranging from 20/10 mg once daily to 30/10 mg twice daily for 10 weeks (130). The trial revealed significant effects, compared with placebo, in reducing agitation in 220 patients with dementia. Another recently developed formulation contains a subclinical quinidine dose and deuterated dextromethorphan (AVP-786) (221). Dextromethorphan was deuterated to improve its pharmacokinetic profile and reduce its first-pass metabolism in the liver (221). After its first results, the FDA agreed to initiate a follow-up, phase III clinical trial (NCT02446132) to evaluate the long-term safety and efficacy of AVP-786 (131). In this extension study, three different dextromethorphan doses are administered twice a day over 52 weeks.

Cannabinoid receptors are also a potential target for agitation treatment due to their neuroprotective effects when employed at not psychoactive doses (222). Δ-9-tetrahydrocannabinol (THC) (132), dronabinol, and nabilone are the most widely investigated cannabinoid receptor agonists in clinical trials (222). While THC failed to show efficacy in reducing agitation or aggressiveness, dronabinol showed effects on nighttime agitation (223). Nabilone appeared to be the most promising and was reported to reduce agitation in a randomized, double-blind, placebo-controlled crossover trial (NCT02351882), although patients experienced significant sedation (133).

Finally, the metabotropic glutamate receptor 2 (mGluR2), N-methyl-d-aspartate receptor (NMDA), muscarinic acetylcholine receptor M4 (M4R), and M1/M4 receptors (M1/M4R) (217) are also promising targets of future intervention. A mGluR2 agonist, LY2979165, is not investigated in one phase II clinical trial. Similarly, the indirect NMDA agonist, SND-51, is in one phase II clinical trial. A selective M4 receptor agonist, HTL0016878, is being tested in one phase I clinical trial. Several compounds, acting on pharmacologically relevant targets, such as the 5-HT6 receptor (5-HT6R) and M1/M4R, have also progressed to advanced preclinical development stages (217). Therefore, one can envision drugs targeting mGluR, NMDA receptors, cannabinoid receptor type 1 (CB1R), M1/M4R, and 5-HT6R to be soon part of the pharmacological armamentarium employed for dementia-related agitation.

## Conclusions

The agitation associated with neurodegenerative diseases can occur at any disease stage and takes a significant toll on patients and caregivers. The symptom also represents a management challenge for clinicians. To date, precise and useful recommendations are still lacking. However, it is safe to say that pharmacological treatments should be implemented as a second choice, whereas non-pharmacological interventions should be person-centered, preferred, and prioritized. Particular attention and research endeavor should be put into the treatment of elderly patients.

## Author Contributions

CC, MR, and LB: paper design and conception. CC, MR, FD, FB, MGR, LF, MD, AD, PA, RS, AG, VF, APi, SS, and LB: manuscript writing. AT, APa, SS, LB, and MO: manuscript revision and editing. CC and MR: figures. All authors agree to be accountable for the content of the work.

## Conflict of Interest

The authors declare that the research was conducted in the absence of any commercial or financial relationships that could be construed as a potential conflict of interest.
